# Lifestyle Modifications and Nutritional and Therapeutic Interventions in Delaying the Progression of Chronic Kidney Disease: A Review

**DOI:** 10.7759/cureus.34572

**Published:** 2023-02-02

**Authors:** Lean Alkhatib, Lorena A Velez Diaz, Samyukta Varma, Arsh Chowdhary, Prachi Bapat, Hai Pan, Geetika Kukreja, Prasannalaxmi Palabindela, Sri Abirami Selvam, Kartik Kalra

**Affiliations:** 1 Internal Medicine, Royal Medical Services, Amman, JOR; 2 Medicine, Universidad de Guayaquil, Guayaquil, ECU; 3 Internal Medicine, Madurai Medical College, Madurai, IND; 4 Nephrology, Smt. Kashibai Navale Medical College and General Hospital, Pune, IND; 5 General Medicine, Smt. Kashibai Navale Medical College and General Hospital, Pune, IND; 6 Pathology, Tianjin University of Chinese Medicine, Tianjin, CHN; 7 Internal Medicine and Hematology/Oncology, Henry Ford Health System, Clinton Township, USA; 8 Internal Medicine, Jennie Stuart Health, Hopkinsville, USA; 9 Internal Medicine, St. Mary Medical Center, Langhorne, USA; 10 Nephrology, Geisinger Medical Center, Danville, USA

**Keywords:** treatment options for diabetic nephropathy, diabetic kidney disease (dkd), chronic kidney disease (ckd), ckd progression, ckd management

## Abstract

Chronic kidney disease (CKD) is a debilitating progressive illness that affects more than 10% of the world's population. In this literature review, we discussed the roles of nutritional interventions, lifestyle modifications, hypertension (HTN) and diabetes mellitus (DM) control, and medications in delaying the progression of CKD. Walking, weight loss, low-protein diet (LPD), adherence to the alternate Mediterranean (aMed) diet, and Alternative Healthy Eating Index (AHEI)-2010 slow the progression of CKD. However, smoking and binge alcohol drinking increase the risk of CKD progression. In addition, hyperglycemia, altered lipid metabolism, low-grade inflammation, over-activation of the renin-angiotensin-aldosterone system (RAAS), and overhydration (OH) increase diabetic CKD progression. The Kidney Disease: Improving Global Outcomes (KDIGO) guidelines recommend blood pressure (BP) control of <140/90 mmHg in patients without albuminuria and <130/80 mmHg in patients with albuminuria to prevent CKD progression. Medical therapies aim to target epigenetic alterations, fibrosis, and inflammation. Currently, RAAS blockade, sodium-glucose cotransporter-2 (SGLT2) inhibitors, pentoxifylline, and finerenone are approved for managing CKD. In addition, according to the completed Study of Diabetic Nephropathy with Atrasentan (SONAR), atrasentan, an endothelin receptor antagonist (ERA), decreased the risk of renal events in diabetic CKD patients. However, ongoing trials are studying the role of other agents in slowing the progression of CKD.

## Introduction and background

Chronic kidney disease (CKD) is a fatal progressive illness and a major cause of mortality worldwide [1,2]. The number of CKD cases has been increasing, with more than 10% of the world population being affected [2]. Diabetes mellitus (DM) and hypertension (HTN) are major risk factors for CKD. However, other risk factors include environmental factors, systemic infections, kidney stones, and nephrotoxins [3]. The main factors implemented in the pathophysiology of kidney injury are immunologic and genetic abnormalities, tissue hypoxia and decreased perfusion, drugs, high glucose, and other substances [4].

**Figure 1:**
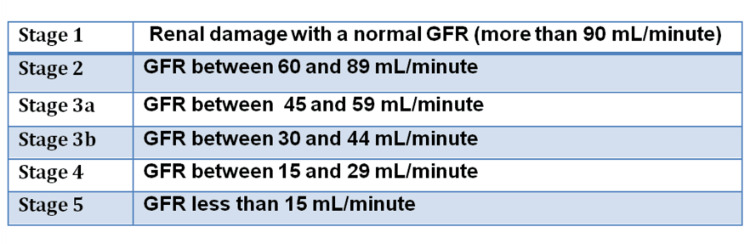
CKD stages CKD, chronic kidney disease; GFR, glomerular filtration rate

CKD patients may present with various symptoms, including generalized weakness, pain, sleep disorders, anxiety, depression, and itching [5]. CKD is diagnosed by meeting one or more of the following for more than a three-month duration: 1) renal structural or functional abnormalities, 2) glomerular filtration rate (GFR) of less than 60 mL/minute/1.73 m^2^, and 3) albuminuria of ≥30 mg per day [6]. There are five stages of CKD consistent with the GFR (Figure 1) [6].

CKD can be complicated by health conditions that can be prevented and treated early in the disease course. Measures to manage CKD include screening high-risk groups (DM, HTN, kidney transplant, and family history of kidney disease) by testing for albuminuria, GFR, urine sediment, and serum creatinine (Cr); treating specific renal diseases; controlling progression; preventing and treating complications; and educating and preparing patients for renal replacement therapy (RRT) [7]. In this literature review, we discussed the roles of nutritional interventions, lifestyle modifications, controlled HTN and DM, and medications in delaying the progression of CKD.

## Review

Lifestyle modifications

Weight Loss

There is a graded causal relationship between central (waist circumference) and general obesity and CKD incidence; a retrospective cohort study observed a significant relationship between high body mass index (BMI) starting from 25.0 kg/m^2^ and the risk of end-stage renal disease (ESRD) [8-10]. A systematic review by Bolignano and Zoccali concluded that weight loss significantly lowered albuminuria (albumin-creatinine ratio of ≥30 mg/g (3 mg/mmol)) and proteinuria, and it was evident predominantly in weight loss following bariatric surgery in patients with DM [11]. However, unintentional weight loss, particularly in malnutrition and muscle wasting, is associated with poor post-kidney-transplant outcomes and an increased risk of death in patients with ESRD [12,13].

Diet

The National Kidney Foundation's Kidney Disease Outcomes Quality Initiative (KDOQI) established that adherence to low-protein diet (LPD) can slow the progression of ESRD and can improve the quality of life in CKD patients [14]. A study conducted by Hahn et al. found that there was a significant lowering of serum urea and serum uric acid in patients on a keto-acid analogue (KA)-supplemented very low-protein diet (VLPD) (0.28-0.43 g/kg/body weight/day) at the end of 15 months when compared to the conventional LPD [15]. However, compared with the normal protein diet, LPD has an uncertain effect on serum creatinine [16]. Other dietary interventions concerning CKD include alternate Mediterranean (aMed) diet and Alternative Healthy Eating Index (AHEI)-2010 [16]. The Chronic Renal Insufficiency Cohort (CRIC) Study reported that participants with the greatest adherence to the aMed diet and AHEI-2010 had a lower risk of progression of CKD and lower all-cause mortality with the strongest results for aMed diet [17]. A low-salt diet (<2.3 g/day) lowers the doubling of serum Cr from the baseline and ESRD. However, the effect of a low-salt diet on the rate of estimated glomerular filtration rate (eGFR) reduction, proteinuria, and all-cause mortality needs further investigation [18].

Physical Activity

The Physical Activity Guidelines for Americans recommends that adults have at least 150 minutes weekly of moderate-intensity physical activity or 75 minutes weekly of vigorous exercise for substantial benefit. Physical activity was controversial in CKD because of the risk of increased proteinuria and renal function impairment. On the contrary, a meta-analysis by Villanego et al. found that low-intensity physical activity improves the quality of life and does not impact renal function [19]. But this study and another meta-analysis by Nakamura et al. did not demonstrate any significant difference between estimated GFR (eGFR) and proteinuria or the progression of CKD in the exercise group with physical activity and the control group with no physical activity [19,20]. Both the exercise and control groups were CKD patients not on dialysis. In another prospective cohort study in stage 3-4 CKD patients by Robinson-Cohen et al., it was found that there was an estimated 0.5% per year of slower decline in eGFR with each 60-minute greater duration of weekly physical activity. The same study also reported a greater reduction in GFR per year in patients with no leisure-time activity compared to patients with guideline-recommended physical activity; there was a 2.8% decline in annual eGFR between patients doing no leisure-time physical activity and patients doing ≥150 minute/week of leisure-time physical activity after adjusting for sociodemographic variables and prevalent diseases [21].

Chen et al. conducted an observational study on patients with stage 3-5 CKD (mean age: 70 years), out of which most patients reported walking as the most common form of exercise. The study found that walking was associated with lower overall mortality and rates of RRT independent of age, renal function, and comorbidities of the patients [22]. The findings from these studies suggest that in older adults, physical activity may slow the rate of decline of kidney function and may be beneficial in lowering the risk of ESRD; there is a dose-dependent association between walking and overall mortality (subdistribution hazard ratio (SHR): 0.83, p=0.04 in patients who walked 1-2 times/week; 0.72, p=0.002 to patients who walked 3-4 times/week; and 0.41, p<0.001 for those walking ≥7 times/week). There is also a dose-dependent reduction in renal replacement therapy with walking (SHR: 0.81 in patients who walked 1-2 times/week and 0.56 for patients who walked 5-6 times/week). However, more studies are warranted to evaluate the minimum level of physical activity required to slow down CKD progression [23].

Smoking

It is difficult to assess the cause and effect of smoking. However, smoking is associated with a worse progression in patients with established CKD. A prospective cohort study conducted in Korea found that smoking is associated with a greater risk of CKD progression during a follow-up of three years. The results were most evident in patients with eGFR of <45 mL/minute/1.73 m^2^ [24]. The same study also reported a higher risk of CKD progression with higher pack years, thus showing a dose-response relationship between smoking and CKD. Similarly, current smokers have a greater risk of CKD progression than people who stopped smoking or have never smoked [25,26]. Some studies also reveal that smoking cessation will delay the progression of CKD when compared to continuous smokers [25].

Limitation of Alcohol Intake

Studies reported that persistent moderate alcohol drinking is not related to the progression of CKD compared to not drinking. However, binge alcohol drinking increases the risk of CKD progression [27,28].

Stress, Working Hours, and Sleep

Stress increases the risk of DM, HTN, and vascular diseases (major risk factors for CKD) by stimulating the sympathetic nervous system and elevating inflammatory cytokines [29]. A cohort study found a significant association between long working hours (>52 hours) and the incidence of CKD. However, further efforts are needed to study the underlying mechanism of this association [30]. Almost 50% of CKD patients have insomnia or impaired sleep quality [31].

Nutritional interventions

Protein consumption is one of the most-often addressed concerns in the dietary therapy of CKD. It has been shown that a high-protein diet, defined as >1.2 g/kg/day, significantly impairs kidney function [32]. LPD provides various benefits for the treatment of CKD patients by reducing nitrogen waste products and decreasing renal strain by lowering intraglomerular pressure, which has a protective impact on the kidneys, particularly in individuals with a diminished reserve of working nephrons [33]. The effects of LPD on kidney physiology are similar to those of renin-angiotensin-aldosterone system (RAAS) blockades. A study showed that LPD had an additive anti-proteinuric effect when combined with RAAS blockades [34].

A caloric intake of 25-35 kcal/kg/day is advised to counterbalance the increased resting energy expenditure caused by inflammation and comorbidities and to maintain a neutral or positive nitrogen balance [35]. According to the 2020 Kidney Disease Outcomes Quality Initiative (KDOQI) recommendations, the advised protein intake for stable patients with non-dialysis stage 3-5 CKD is 0.55-0.60 g/kg/day, which may be decreased to 0.28-0.43 g/kg/day if supplemented with 7-15 g/day of keto-acid analogues (KA) and necessary essential amino acids, and patients with stage 3-5 CKD dietary protein restriction combined with KA supplementation likely have an overall positive effect on RRT/renal survival. In addition, a moderate sodium restriction (2.3 g/day) is indicated for the treatment of CKD patients to promote improved volume control, blood pressure (BP) reduction, and proteinuria reduction in conjunction with current pharmaceutical strategies [13].

Acidosis is one of the major risk factors for CKD progression. A study showed that fruits and vegetables are an option for oral alkali that may minimize the risk for volume retention and hypertension associated with bicarbonate supplementation. In this study, dieticians recommended 2-4 cups of fruits and vegetables per day [36]. Another case-control study by Di Iorio et al. showed that a VLPD rich in vegetables and fruits and low in protein supplemented with essential amino acids and keto-acid analogues of nonessential amino acids significantly decreased net endogenous acid production (NEAP) by 53% at six months and 67% at 12 months and potential renal acid load (PRAL) by 120% at six months and 138% after 12 months [37].

Blood sugar control

The kidneys significantly impact glucose homeostasis via glucose glomerular filtration, reabsorption through sodium-glucose cotransporters (SGLT), and generation [38]. Hemoglobin A1C is the gold standard for glycemic assessment in CKD patients, but it is affected by hematologic factors. Glycated albumin has been proposed as a marker of glycemic control in CKD patients. However, it is affected by proteinuria, and further studies are needed to evaluate its role in diabetic CKD [38]. Continuous glucose monitoring (CGM) device is a minimally invasive method that can monitor glycemic control in CKD patients. It is interpreted as the following: 1) time in range, the time spent within the target blood sugar range (70-180 mg/dL); 2) time above range, the time spent above the target (level 1: 181-250 mg/dL; level 2: >250 mg/dL); and 3) time below target, the time spent below the target (level 1: 54-69 mg/dL; level 2: <54 mg/dL) [38]. Two retrospective cohort studies found a higher risk of death associated with A1C levels of <6%-7% or >9%. The Action to Control Cardiovascular Risk in Diabetes (ACCORD) trial found a higher risk of death in CKD patients with strict A1C control (6.7%) compared to standard control (A1C: 7.5%) [38].

A shift in the treatment of CKD associated with type 2 diabetes mellitus (T2DM) has been made since the first clinical trial showing the benefits of sodium-glucose cotransporter-2 (SGLT2) inhibitors was published in 2015. Before this discovery, glycemic and blood pressure (BP) control were the mainstay for delaying the progression of CKD and cardiovascular diseases associated with T2DM. SGLT2 inhibitors were found to reduce the major risk of cardiovascular events and the advancement of chronic kidney disease in T2DM patients [39]. Clinical studies have shown that SGLT2 inhibitors have diverse pleiotropic effects, including modulating neurohormones such as the RAAS, increasing hematocrit, altering energy substrate use, and attenuating systemic inflammation and oxidative stress, all of which are implicated in the cardiovascular and kidney protective effects of SGLT2 inhibitors [39]. There is limited data regarding the risk factors related to kidney disease in patients with newly diagnosed T2DM. The overall rate of CKD progression is relatively high, and hypoalbuminemia and elevated albuminuria are associated with kidney failure progression in newly diagnosed T2DM patients [40].

A prospective cohort study established a relationship between overhydration (OH), measured using bioimpedance assay (BIA), and CKD progression in patients with T2DM. The study found that OH is an independent risk factor for CKD progression in patients with T2DM. The authors hypothesized that OH was a predictor, while pigment epithelium-derived factor (PEDF) was a modifiable risk factor for the progression of CKD [41]. Patients with T2DM in the highest tertile of OH and relative OH (OH/extracellular water of >7%) were positively associated with CKD progression (hazard ratio (HR): 1.45 (95% confidence interval (CI): 1.14-1.85), p=0.003; HR: 1.29 (95% CI: 1.05-1.59), p=0.017). There were positive associations between PEDF and CKD progression (β=1.10; p=0.001) and between OH and CKD progression (β=0.21; p=0.036). OH remained positively associated with CKD progression mediated by PEDF [41].

Blood pressure control

Uncontrolled HTN adversely affects the prognosis in patients with CKD; therefore, strict BP control is mandatory [42]. Hypertension is the most common complication associated with CKD. Its prevalence is nearly 100% in stage 4-5 CKD [43]. Reducing BP in patients with CKD has two primary goals: 1) reducing the probability of cardiovascular events and subsequent mortality and 2) preventing the progression of CKD and the need for dialysis or renal transplant [44].

Patients with CKD have a high prevalence of nocturnal hypertension [45]. Higher nocturnal BP is detrimental and can lead to a higher risk of cardiovascular outcomes. From day to night, there is a normal reduction in nocturnal BP. Patients who experience that decline in nocturnal BP are called dippers, but patients who do not are called non-dippers. Patients with a non-dipper variation are at a higher cardiovascular risk than dippers [46]. Non-dippers are common among CKD patients [47]. Higher nighttime sympathetic nervous system activity and the circadian rhythm of urinary sodium exertion are the suggested mechanisms of elevated nocturnal BP [45]. Further studies are needed to evaluate nocturnal electrolyte excretion, sympathetic nervous system activity, the splitting of 24-hour urinary collection, and their relationship to dipping status [45].

The Systolic Blood Pressure Intervention Trial (SPRINT) have shown that intensive BP control (systolic BP of less than 120) in individuals with an increased cardiovascular risk and mild-to-moderate CKD significantly decreases the risk of cardiovascular events and all-cause mortality. However, the intensive BP management effect on CKD progression, acute kidney injury (AKI), and CKD incidence needs further evaluation [48]. In non-dialysis CKD patients, the Kidney Disease: Improving Global Outcomes (KDIGO) 2021 guidelines recommend a systolic BP of less than 120, while the 2017 American College of Cardiology (ACC)/American Heart Association (AHA) guidelines recommend a systolic BP of less than 130 [49].

**Figure 2:**
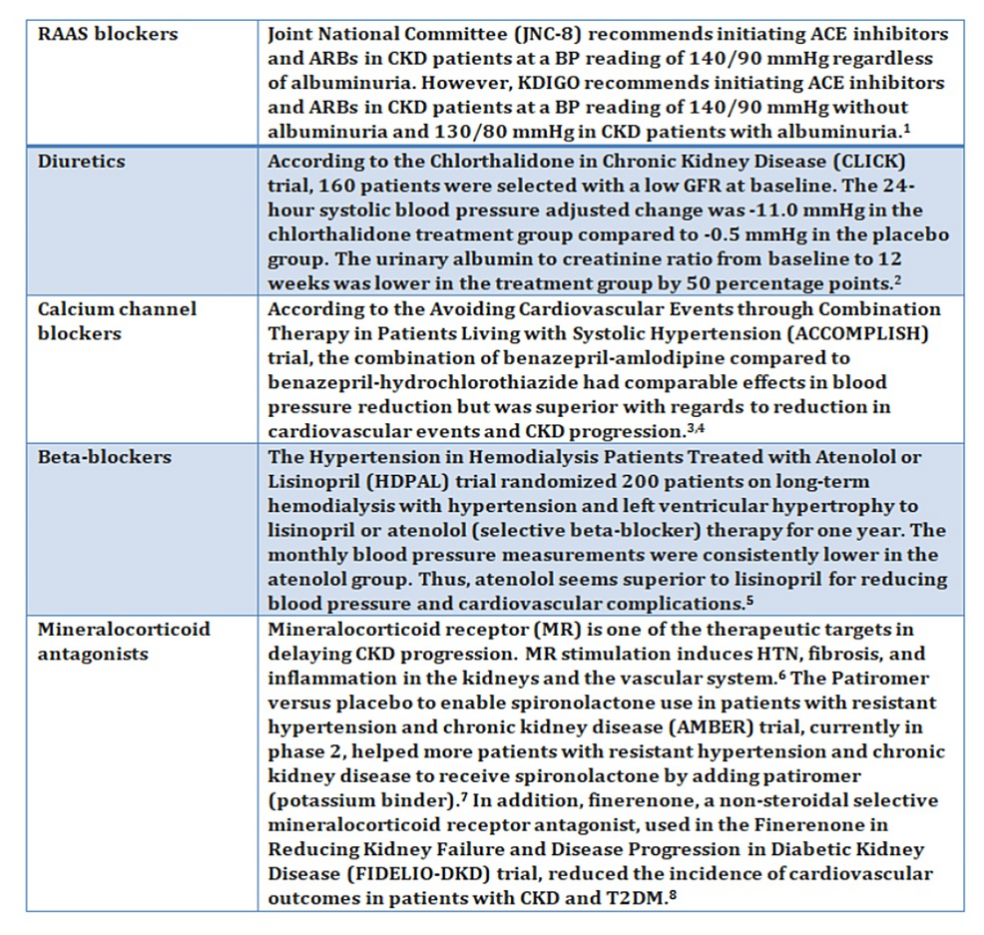
Pharmacological management of hypertension in CKD patients 1: [50], 2: [51], 3: [52], 4: [53], 5: [54], 6: [55], 7: [56], and 8: [57] RAAS, renin-angiotensin-aldosterone system; ACE, angiotensin-converting enzyme; ARBs, angiotensin receptor blockers; CKD, chronic kidney disease; BP, blood pressure; KDIGO, Kidney Disease: Improving Global Outcomes; GFR, glomerular filtration rate; HT, hypertension; T2DM, type 2 diabetes mellitus

Figure 2 shows the pharmacological management of hypertension in CKD patients.

Medical management

**Figure 3:**
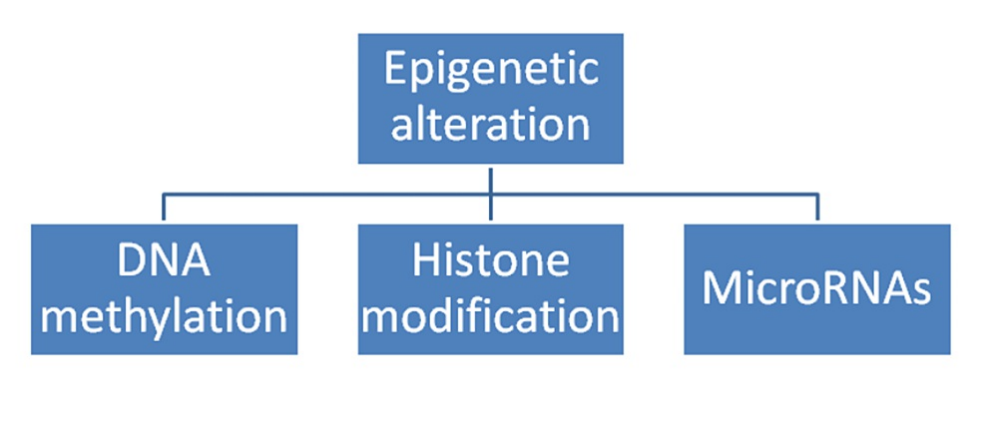
Epigenetic modifications related to CKD progression CKD: chronic kidney disease

**Figure 4:**
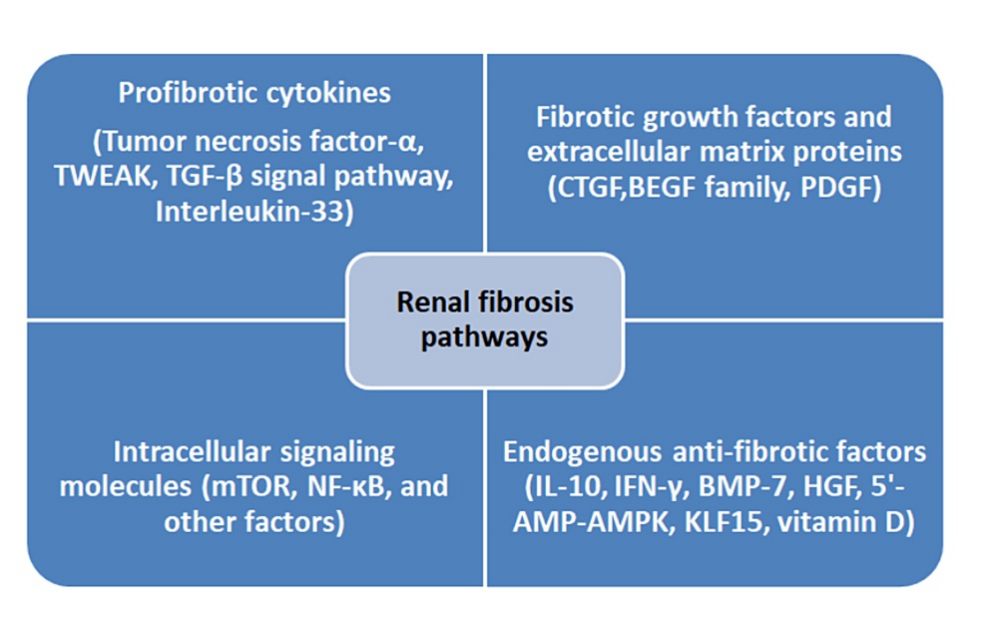
Renal fibrosis pathways TWEAK, tumor necrosis factor-like weak inducer of apoptosis; TGF, transforming growth factor; CTGF, connective tissue growth factor; EGF, epidermal growth factor; PDGF, platelet-derived growth factor; mTOR, mammalian target of rapamycin; NF-κB, nuclear factor kappa-light-chain-enhancer of activated B cells; IL-10, interleukin 10; IFN, interferon; BMP-7, bone morphogenetic protein 7; HGF, hepatocyte growth factor; 5'-AMP, 5'-adenosine monophosphate; AMPK, adenosine monophosphate-activated protein kinase; KLF15, Kruppel-like factor-15

CKD is a progressive illness that leads to the loss of nephrons. Current therapies for CKD aim to control its progression by targeting epigenetic alterations (Figure 3), fibrosis, and inflammation [58]. Fibrosis is mediated by collagen-producing myofibroblasts and enzymes responsible for collagen degradation [58]. In addition, multiple inflammatory cells and cytokine signalling pathways interact and lead to renal fibrosis (Figure 4) [59].

Renin-Angiotensin System (RAS) Inhibitors

Angiotensin-converting enzymes (ACEIs) and angiotensin receptor blockers (ARBs) are the first-line medications in proteinuric CKD patients. They delay the progression of CKD and lower the risk of cardiovascular outcomes in diabetic and non-diabetic CKD patients. Also, ACEIs were found to reduce all-cause mortality in these patients [60]. However, their role in advanced CKD (stages 4 and 5) is still under investigation [60]. A meta-analysis of randomized clinical trials held by Zhang et al. found that ACE inhibitors are superior to ARBs in delaying the progression of CKD to ESRD, reducing cardiovascular events, cardiovascular death, and all-cause death in non-dialysis patients with stage 3-5 CKD, while in diabetic advanced CKD patients, ARBs were superior to ACEIs in reducing the progression of CKD and cardiovascular outcomes but inferior to ACEIs in lowering all-cause mortality [61]. Symptomatic hypotension, hyperkalemia, and vulnerability to AKI as side effects of RAS inhibitors are more commonly seen in patients with advanced CKD [60].

SGLT2 Inhibitors

SGLT2 inhibitors have a nephroprotective effect by reducing albuminuria. They increase the delivery of sodium to distal tubules, inhibit tubuloglomerular feedback, and minimize intraglomerular pressure and albuminuria via afferent vasoconstriction. Also, SGLT2 inhibitors have an anti-inflammatory effect by decreasing profibrotic factors, interleukin-6, and nuclear factor kappa-light-chain-enhancer of activated B cells (NF-κB). This drug class inhibits the progression of albuminuric CKD in diabetic and non-diabetic patients, including those with heart failure [62]. However, their effect on kidney transplant patients and those with ESRD warrants further investigation [62]. In diabetic kidney disease (DKD) patients, SGLT2 inhibitors were found to decrease the risks of all-cause mortality, renal events (declining GFR, worsening creatinine, ESRD, or death from renal or cardiovascular causes), hospitalization for heart failure (HHF), and major adverse cardiovascular events (MACE) including myocardial infarction (MI), stroke, or cardiovascular mortality [63]. Avoiding SGLT2 inhibitors is advised in patients with recurrent urinary tract infections (UTIs) and genital infections. Other risks associated with the use are Fournier gangrene, euglycemic diabetic ketoacidosis (euDKA), and fractures [64]. SGLT2 inhibitors were found to decrease the risk of AKI in most cases. However, this does not preclude that SGLT2 inhibitors can exacerbate AKI in volume-depleted patients [65]. SGLT2 inhibitors may induce an initial acute decline in eGFR. Subsequently, this reduction is attenuated with the continuation of this class of drugs. The initial drop in eGFR of 30% following the initiation of SGLT2 inhibitors is attributed to the intraglomerular pressure reduction. It is possible to distinguish this initial eGFR dip from AKI in patients taking SGLT2 inhibitors by following up with kidney function over 2-4 weeks [66].

Finerenone

Mineralocorticoid receptor (MR) gene expression is involved in electrolyte balance and fluid homeostasis. Also, MR is over-activated in patients with chronic diseases such as CKD and T2DM. This over-activation leads to increased inflammation, fibrosis, and end-organ damage. In contrast to spironolactone and eplerenone as steroidal MR blockers, finerenone is a nonsteroidal MR blocker. This nonsteroidal structure reduces inflammation and fibrosis by inhibiting the recruitment of transcriptional factors. In comparison to spironolactone, finerenone causes less hyperkalemia [67]. According to the Finerenone in Reducing Kidney Failure and Disease Progression in Diabetic Kidney Disease (FIDELIO-DKD) trial, a phase III double-blinded randomized placebo-controlled trial, finerenone was found to lower the risk of ESRD, eGFR decline, HHF, nonfatal MI, and cardiovascular mortality in patients with CKD and T2DM [68].

Pentoxifylline (PTF)

Pentoxifylline (PTF) is a nonspecific phosphodiesterase inhibitor demonstrating a kidney-protective effect through its anti-inflammatory effect; PTF reduces high-sensitivity C-reactive protein (CRP), tumor necrosis factor-alpha (TNF-α), and fibrinogen levels. Also, it improves kidney function by increasing the eGFR at 12 months. As a result, PTF decreases the progression of CKD to ESRD, particularly in albuminuric patients, regardless of the presence of diabetes mellitus and reduces the risk of cardiovascular mortality in CKD patients [69]. On the other hand, gastrointestinal complaints are the most common side effects of PTF, but they are more commonly found in patients whose PTF dosages are not adjusted according to their kidney function. However, such side effects are self-limited and improve by adjusting the dose of PTF [70].

Atrasentan (Completed Trials)

Endothelin receptor antagonist (ERA) protects the kidney via its antihypertensive, anti-albuminuric, anti-inflammatory, and anti-fibrotic effects. Also, it reduces hyperfiltration and endothelial injury while increasing podocyte number. This class of drugs demonstrates its anti-inflammatory effect via reducing NF-κB, transforming growth factor-β (TGF-β), and extracellular matrix (ECM) accumulation. The anti-albuminuric impact is related to increasing the number of podocytes and reducing the permeability of albumin (endothelin-1 (ET-1) stimulates angiotensin II production, thus increasing albumin permeability) [71]. According to the Study Of Diabetic Nephropathy With Atrasentan (SONAR) study, a phase III double-blinded placebo-controlled randomized study, atrasentan decreased the risk of renal events in diabetic CKD patients. Nonselective endothelin receptor antagonists increase the risk of heart failure. However, less significant fluid retention was seen among patients receiving a short course with low doses of the selective endothelin A receptor antagonist (atrasentan) [72].

Pirfenidone (Ongoing Trials)

Pirfenidone is an anti-fibrotic agent that has both anti-fibrotic (via inhibiting the expression of TGF-β1) and anti-inflammatory (via tumor necrosis factor (TNF) expression) effects. Those effects were seen in animal models. Pirfenidone was approved for the management of idiopathic pulmonary fibrosis (IPF). It is renally excreted, and the Food and Drug Administration (FDA) recommends using it cautiously when creatinine clearance is <80 mL/minute. Also, it is contraindicated in dialysis patients. Pirfenidone has side effects, including transaminitis, gastrointestinal symptoms, and photosensitivity. Currently, the Trial of Pirfenidone to Prevent Progression in Chronic Kidney Disease (TOP-CKD) is an ongoing phase II trial that will be finished in December 2024. It involves 200 patients and aims to study pirfenidone's role in CKD [73].

## Conclusions

CKD is a growing worldwide health burden. As a result, active efforts have been made to study CKD progression; this literature review summarizes the role of DM and HTN control, nutritional and therapeutic interventions, and lifestyle modification, including weight loss, diet, physical activity, smoking, and alcohol, in delaying the progression of CKD. Obesity, smoking, binge alcohol drinking, uncontrolled DM, and hypertension tend to worsen CKD. However, RAAS blockade, SGLT2 inhibitors, pentoxifylline, and and finerenone are the currently approved medications for managing CKD progression. The role of other agents is being studied through completed and ongoing clinical trials.
